# Screening for differentially expressed genes between left- and right-sided colon carcinoma by microarray analysis

**DOI:** 10.3892/ol.2013.1414

**Published:** 2013-06-18

**Authors:** HONG ZHU, TIAN-CONG WU, WEI-QIONG CHEN, LI-JUN ZHOU, YUE WU, LIANG ZENG, HAI-PING PEI

**Affiliations:** 1 Department of Oncology, Xiangya Hospital, Central South University, Changsha, Hunan 410008, P.R. China; 2 Department of Pathology, Hunan Tumor Hospital, Changsha, Hunan 410013, P.R. China; 3 Department of Gastrointestinal Surgery, Xiangya Hospital, Central South University, Changsha, Hunan 410008, P.R. China

**Keywords:** left-sided colon carcinoma, right-sided colon carcinoma, cDNA microarray, differential gene expression

## Abstract

Left-sided colon carcinoma (LSCC) and right-sided colon carcinoma (RSCC) differ in their genetic susceptibilities to neoplastic transformation. The present study identified 11 genes that were differentially expressed in LSCC and RSCC by expression profiling with microarray analysis. Compared with RSCC, the human genes for L-lactate dehydrogenase B chain (LDHB), cyclin-dependent kinase 4 inhibitor D (CDKN2D), phosphatidylinositol-4-phosphate-3-kinase C2 domain-containing subunit α (PI3KC2α), protocadherin fat 1 (FAT; a human protein that closely resembles the Drosophila tumor suppressor, fat) and dual specificity protein phosphatase 2 (DUSP2) were upregulated in LSCC. By contrast, genes for ubiquitin D (UBD), casein kinase-1 binding protein (CK1BP), synaptotagmin-13 (SYT1), zinc finger protein 560 (ZNF560), pleckstrin homology domain-containing family B member 2 (PLEKHB2) and IgGFc-binding protein (FCGBP) were downregulated in LSCC compared with RSCC. A quantitative polymerase chain reaction (qPCR) analysis revealed that the mRNA levels of *UBD* and *CK1BP* in LSCC were significantly lower compared with those in RSCC (P=0.033 and P= 0.005, respectively), whereas the mRNA levels of *LDHB* and *CDKN2D* in LSCC were significantly higher compared with those in RSCC (P=0.008 and P=0.017, respectively). Western blot and immunohistochemical analyses demonstrated that the expression of CDKN2D in LSCC was significantly higher compared with that in RSCC, while the expression of UBD in LSCC was significantly lower compared with that in RSCC. The present study provides important insights into the understanding of the molecular genetic basis for the different biological behaviors observed between LSCC and RSCC. These insights may therefore serve as a basis for the identification of novel colon cancer markers and therapeutic targets.

## Introduction

Colon cancer is a significant cause of cancer-related morbidity and mortality, and is the third most fatal malignancy worldwide (1). In China and other economically developing countries, colon cancer incidence rates have increased over the past 20 years; most likely due to changes in the environment, individual lifestyle and nutritional habits (2). In certain high-prevalence regions, colon cancer has become the second leading cause of cancer-related mortality (3). It has been suggested that there are two distinct categories of colon cancer (CRC), i.e., CRC that is proximal or distal to the splenic flexure (4). A number of studies have demonstrated that right-sided (proximal) and left-sided (distal) colon tumors differ in their genetic susceptibilities to neoplastic transformation (5–9). Significant differences have been observed to exist between left-sided colon carcinoma (LSCC) and right-sided colon carcinoma (RSCC), with regard to epidemiological, biological and clinical data concerned with carcinogenesis and survival (10–13). Christodoulidis *et al* reported that the size of colonic tumors was significantly greater in RSCC compared with LSCC and that LSCC patients had a significantly improved overall 5-year survival rate compared with RSCC patients (10). Wray *et al* reported that LSCC presented at an earlier stage, had a lower tumor grade and independently decreased colorectal cancer-specific mortality compared with RSCC (11). Papagiorgis *et al* reported that RSCC had higher severity in terms of stage and grade compared with LSCC (13). However, the molecular genetic basis for the different biological behaviors between LSCC and RSCC remains unclear. Using cDNA microarray analysis, the present study explored the differentially expressed genes of LSCC and RSCC.

## Materials and methods

### Patients

From June 2007 to December 2008, 100 Han Chinese patients diagnosed with sporadic colon adenocarcinoma (LSCC, n=50; RSCC, n=50) were recruited from the Department of General Surgery of Xiangya Hospital, Central South University (Changsha, China). All patients received complete resection of the tumor, without pre-operative chemotherapy or radiotherapy. The baseline characteristics of the patients are listed in Table I. The study was approved by the Ethical Committee of Xiangya Hospital, Central South University. Informed consent was obtained from all participants.

### Reagents

The Nanjing University 22K Human Genome Array gene chip was purchased from CapitalBio Corp. (Beijing, China). The gene chip contained 21,522 70-mer oligo-nucleotide DNAs, each representing a human gene transcript. Among the 21,522 oligonucleotide DNAs, 21,329 were from the Human Genome Oligo Set, Version 2.1 (Eurofins MWG Operon, Huntsville, AL, USA) and the remaining 193 were synthesized by CapitalBio Corp. The anti-cyclin-dependent kinase 4 inhibitor D (CDKN2D) monoclonal (sc-71810) and goat anti-human ubiquitin D (UBD; sc-51082) antibodies were purchased from Santa Cruz Biotechnology, Inc. (Santa Cruz, CA, USA).

### RNA isolation and microarray procedures

The total RNA was extracted from samples using TRIzol reagent (Invitrogen Life Technologies, Carlsbad, CA, USA), and purified using the NucleoSpin RNA Clean-up kit (Macherey-Nagel GmbH and Co., KG, Düren, Germany). The total RNA was then transcribed into double stranded cDNA with a cDNA Synthesis kit obtained from Promega Corporation (Madison, WI, USA), and purified with a polymerase chain reaction (PCR) NucleoSpin Extract II kit (Promega Corporation). The double stranded cDNA was transcribed *in vitro*, compounded into cRNA, purified and then labeled with Cy5-dCTP (Amersham Pharmacia Biotech, Inc., Piscataway, NJ, USA) by the Klenow enzyme (Takara Bio, Inc., Otsu, Japan). Hybridization was performed at 42°C with the Nanjing University 22K Human Genome Array gene chip (CapitalBio Corp.).

### Chip scan and data analysis

The chip images were scanned using a LuxScan 10K-A double-channel laser scanner (CapitalBio Corp.). The signals referred to the unified data of the light intensity that were detected by the scanner and analyzed with the CapitalBio SpotData Pro 3.0 Microarray Image Analysis software (CapitalBio Corp.). The image signals were transmitted as digital signals, and then the data contained on the chips were normalized by the Lowess method (14).

### Quantitative (q)PCR

The total RNA were prepared using TRIzol reagent (Invitrogen Life Technologies) followed by purification with the Turbo DNA-*free* system (Ambion, Inc., Austin, TX, USA). The cDNA was synthesized using SuperScript II Reverse Transcriptase (Invitrogen Life Technologies). qPCR was performed using the LightCycler thermal cycler system (Roche Diagnostics GmbH, Mannheim, Germany) with the SYBR Green I kit (Roche Diagnostics GmbH), according to the manufacturer’s instructions. The results were normalized against those of the housekeeping gene, glyceraldehyde-3-phosphate dehydrogenase (*GAPDH*), in the same sample. The following primer sequences were used: *CDKN2D* forward, 5′-CTCGCCGTCCTCCGGCTGAC-3′ and reverse, 5′-AGCATGTCGACACTGGCGGC-3′; casein kinase-1 binding protein (*C20orf35*) forward, 5′-CCCTTTCCTCCTCTTCATCC-3′ and reverse, 5′-CCCTTT CCTCCTCTTCATCC-3′; L-lactate dehydrogenase B chain (*LDHB*) forward, 5′-TCCCGTGTCAACAATGGTAA-3′ and reverse, 5′-CCCACAGGGTATCTGCACTT-3′; *UBD* forward, 5′-GGCACCTCCTCCAGGTGCGAA-3′ and reverse, 5′-CAACACCCCATGCCCAGGGTG-3′; *GAPDH* forward, 5′-GTCAGTGGTGGACCTGACCT-3′ and reverse, 5′-TGCTGTAGCCAAATTCGTTG-3′. Each sample was repeated in triplicate. The results are expressed as the mean ± standard deviation.

### Western blot analysis

Immunoblotting was performed with the respective antibodies. Briefly, extracted tumor tissues were homogenized and lysed in 0.1% Nonidet P-40 lysis buffer (0.1% Nonidet P-40; 50 mM Tris-HCl, pH 7.4; 150 mM NaCl; and 1 mM EDTA). Equal quantities of protein for each sample were separated by 10% sodium dodecyl sulfate (SDS)-polyacrylamide gels and blotted onto a polyvinylidene difluoride microporous membrane (Millipore, Billerica, MA, USA). The membranes were incubated for 1 h with a 1:1,000 dilution of anti-CDKN2D monoclonal antibody (sc-71810) or goat anti-UBD antibody (sc-51082) (Santa Cruz Biotechnology, Inc.), and then washed and revealed using secondary antibodies with horseradish peroxidase conjugate (1:5,000; 1 h). The peroxidase was revealed with an enhanced chemiluminescence (ECL) kit obtained from GE Healthcare Lifesciences (San Francisco, CA, USA). The proteins were quantified prior to being loaded onto the gel, and the equal loading of extracts was verified by an analysis of Ponceau coloration.

### Immunohistochemistry

Paraffin-embedded tumor tissues were examined for CDKN2D or UBD expression. The immunostaining for CDKN2D and UBD was performed by utilizing the streptavidin-biotin-peroxidase method, according to the manufacturer’s instructions (Beijing Zhongshan Golden Bridge Biotechnology Co., Ltd., Beijing, China). Briefly, 4-*μ*m sections of paraffin-embedded specimens were de-paraffinized in xylene, hydrated in a degraded series of ethanol and heated in 0.01 M citrate buffer for 10 min in a microwave oven. Subsequent to cooling for 20 min and washing in PBS, endogenous peroxidase was blocked with methanol containing 0.3% hydrogen peroxide for 30 min, followed by incubation with phosphate buffered saline (PBS) for 30 min. Next, the sections were incubated with the anti-CDKN2D or -UBD antibody at a dilution of 1:150, and stained using the avidin-biotin complex method. Coloration was developed by 3,3′-diaminobenzidine (DAB) containing H_2_O_2_, and the sections were counterstained with hematoxylin. Two pathologists, blinded to the clinical and pathological data, independently examined the slides by randomly selecting 10 high-power (x400) view fields in each sample and scoring the gene expression in the tumor cells, as previously described (15). Briefly, each tumor sample was administered a score according to the intensity of the nucleic or cytoplasmic staining (0, no staining; 1, weak staining; 2, moderate staining; and 3, strong staining) and the extent of stained cells (0%, 0; 1–10%, 1; 11–50%, 2; 51–80%, 3; and 81–100%, 4). The extent of stained cells was classed as either negative, focally positive or diffusely positive, corresponding to the 0, 1–80 and 81–100% stained areas, respectively. The final immunoreactive score was determined by multiplying the staining intensity scores by the extent of staining scores, with a minimum score of 0 and a maximum score of 12. Scores of 0–3 constituted negative staining, while scores of 4–12 indicated positive staining (15).

### Statistical analysis

Statistical analyses were performed with SPSS for Windows 10.0 (SPSS, Inc., Chicago, IL, USA). Numerical data are presented as the mean ± standard deviation. Comparisons were performed with the Student’s t-test, following the assessment of normality and equality of variances. Categorical variables were compared using the χ^2^ test. P<0.05 was used to indicate a statistically significant difference.

## Results

As demonstrated in Table I, no significant differences were observed between the LSCC and RSCC patients in terms of the baseline characteristics, including age, gender, TNM staging, tumor cell differentiation, tumor size, tumor invasion and rate of lymph node or liver metastasis. The results indicated that the LSCC and RSCC groups were comparable at baseline.

Gene expression profiling of LSCC and RSCC was established using the Nanjing University 22K Human Genome Array gene chip (CapitalBio Corp.), which has been employed in previous studies for microarray analysis (16–7). The screening criteria for differentially expressed genes were as follows: score difference, ≥2; LSCC/RSCC fold change, 0.5–2; and number of biological replicates, ≥3. As demonstrated in Table II, 11 genes were identified to be differentially expressed between LSCC and RSCC. Compared with RSCC, genes for LDHB, CDKN2D, phosphatidylinositol-4-phosphate-3-kinase-C2 domain-containing subunit alpha (PI3KC2α), protocadherin fat 1 (FAT) and dual specificity protein phosphatase 2 (DUSP2) were upregulated in LSCC. By contrast, genes for UBD, C20orf35, synaptotagmin-13 (SYT1), zinc finger protein 560 (ZNF560), pleckstrin homology domain-containing family B member 2 (PLEKHB2) and IgGFc-binding protein (FCGBP) were downregulated in LSCC, compared with RSCC.

**Figure 1.**
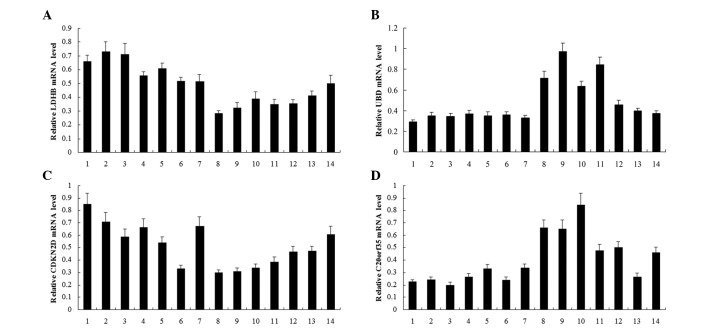
mRNA levels of differentially expressed genes in left-sided colon carcinoma (LSCC) and right-sided colon carcinoma (RSCC). Quantitative polymerase chain reaction (qPCR) was performed to examine the mRNA levels of differentially expressed genes in LSCC (sample nos. 1–7) and RSCC (sample nos. 8–14). (A) L-lactate dehydrogenase B chain (*LDHB*); (B) ubiquitin D (*UBD*); (C) cyclin-dependent kinase 4 inhibitor D (*CDKN2D*); (D) casein kinase-1 binding protein (*C20orf35*). The mRNA level of each differentially expressed gene was normalized against that of the housekeeping gene, glyceraldehyde-3-phosphate dehydrogenase (*GAPDH*), in the same sample. Each sample was repeated in triplicate and results are expressed as the mean ± standard deviation.

**Figure 2.**
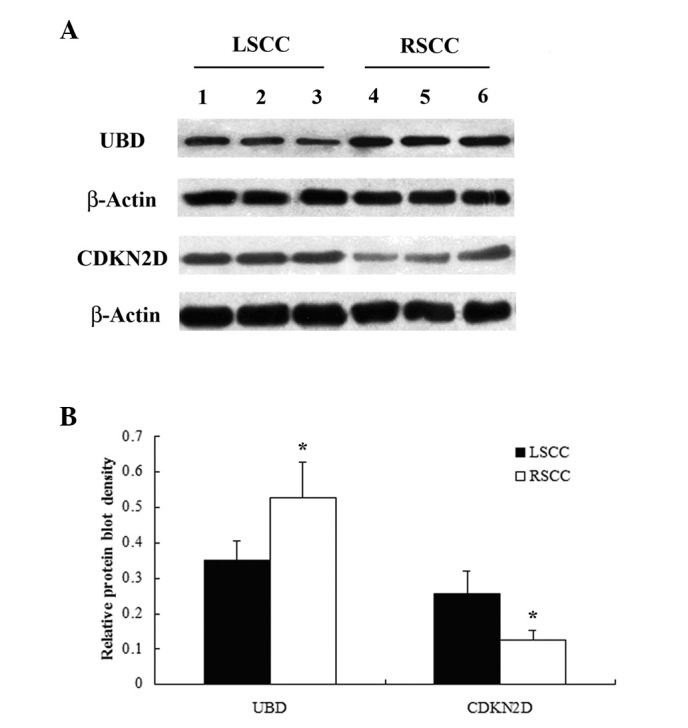
Western blot analysis of ubiquitin D (UBD) and cyclin-dependent kinase 4 inhibitor D (CDKN2D) expression in left-sided colon carcinoma (LSCC) and right-sided colon carcinoma (RSCC). (A) LSCC and RSCC tissue lysates were subject to western blot analysis for UBD and CDKN2D expression. β-actin blotting was used as a loading control. (B) UBD, CDKN2D and β-actin blots were measured by densitometry. The densities of the UBD and CDKN2D blots were normalized against that of β-actin, to obtain a relative UBD or CDKN2D blot density. ^*^P<0.05 compared with LSCC.

**Figure 3.**
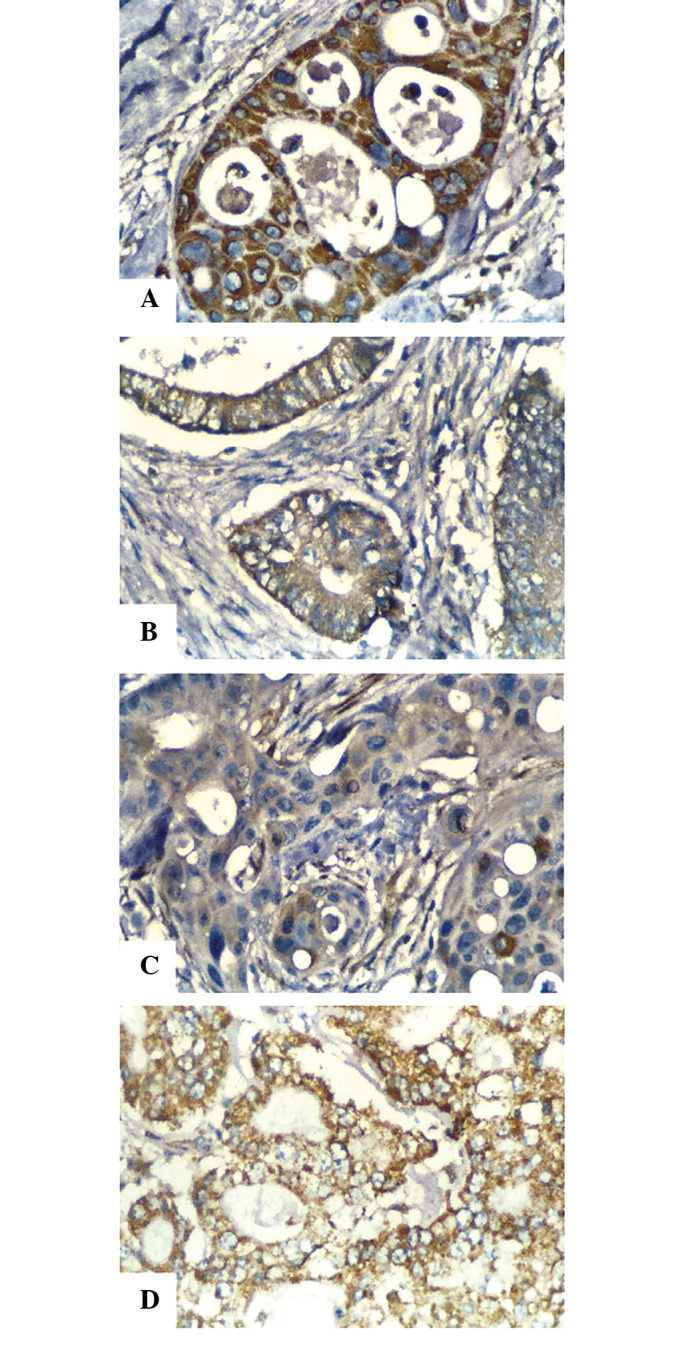
Immunohistochemical detection of ubiquitin D (UBD) and cyclin-dependent kinase 4 inhibitor D (CDKN2D) expression in left-sided colon carcinoma (LSCC) and right-sided colon carcinoma (RSCC). Immunohistochemical analyses were performed to determine (A and B) CDKN2D and (C and D) UBD expression in (A and C) LSCC and (B and D) RSCC. Sections were stained using the avidin-biotin complex method. The coloration was developed with 3,3′-diaminobenzidine (DAB), and the sections were then counterstained with hematoxylin. Positive staining for either CDKN2D or UBD appeared brown. Magnification, x400.

As demonstrated in Fig. 1, qPCR revealed that the mRNA levels of *UBD* and *C20orf35* in LSCC were significantly lower than those in RSCC (P= 0.033 and P= 0.005, respectively), while those of *LDHB* and *CDKN2D* were significantly higher in LSCC than those in RSCC (P=0.008 and P=0.017, respectively), thus confirming the results of the microarray analysis. The western blot analyses demonstrated that the protein level of CDKN2D in LSCC was significantly higher than that in RSCC (P=0.041), while that of UBD in LSCC was significantly lower compared with that in RSCC (P=0.029) (Fig. 2). The immunohistochemical analyses revealed that the positive staining rate of CDKN2D in LSCC was significantly higher than that in RSCC (P= 0.016), while that of UBD in LSCC was significantly lower compared with that in RSCC (P=0.007; Table III; Fig. 3).

## Discussion

Significant differences exist between LSCC and RSCC, with regard to epidemiological, biological and clinical data concerned with carcinogenesis and survival (10–13). A number of studies have suggested that LSCC and RSCC differ in their genetic susceptibilities to neoplastic transformation (5–9). Using expression profiling with microarray analysis, the present study identified 11 genes that were differentially expressed in LSCC and RSCC.

Compared with RSCC, five genes were upregulated in LSCC; *LDHB*, *CDKN2D*, *PI3KC2α*, *FAT* and *DUSP2*. LDHB is an important enzyme in sugar metabolism. Griffini *et al* proposed that LDH may be involved in increasing anaerobic glycolysis in the metastatic foci of CRC in the liver, suggesting that the LDH activity may reflect the status of tumor metabolism (18). In the present study, LSCC demonstrated higher *LDHB* expression than RSCC, implicating that the left and right side of the colon differ in their tumor metabolic activity, particularly with regard to anaerobic glucose metabolism. An RSCC is typically larger and has a poorer survival outcome compared with LSCC, which may be due to the fact that symptoms such as bleeding and pain occur later on in RSCC (10). CDKN2D has been demonstrated to induce tumor cell apoptosis through the cyclin D-CDK4/6-INK4-Rb pathway (19). The present study identified that LSCC had increased CDKN2D expression compared with RSCC, suggesting an additional mechanism for the differences in tumor size and survival outcome between LSCC and RSCC.

Compared with RSCC, six genes were downregulated in LSCC; *UBD*, *C20orf35, SYT13, ZNF560*, *PLEKHB2* and *FCGBP*. Yan *et al* demonstrated that UBD may contribute to the progression of colon carcinogenesis and function as a novel prognostic indicator that may predict tumor recurrence in stage II and III patients following curative surgery (20). The results of the present study revealed that RSCC had higher *UBD* expression than LSCC, suggesting that RSCC may have a poorer prognosis compared with LSCC, which is concordant with the results of a study by Christodoulidis *et al* (10). Studies have investigated the association between *FCGBP* expression and the presence of tumors. O’Donovan *et al* revealed that while the *FCGBP* gene was constitutively expressed in normal thyroid tissue, its expression was significantly decreased in papillary and follicular thyroid carcinomas (21). The correlation between the expression of *FCGBP*, *C2*, *FAT* or *DUSP2* and LSCC or RSCC remains unclear. Further studies are required to uncover the role of these genes in the pathogenesis and progression of LSCC and RSCC. Thus, the present results not only confirm those of previous studies, but also suggest potential targets for future studies on the pathogenesis and progression of colon cancer, which may serve as a basis for the identification of novel colon cancer markers and therapeutic targets. In addition, it may be useful to explore the potential interactions between the differentially expressed genes and well-studied oncogenes or tumor suppressor genes, such as V-Ki-ras2 Kirsten rat sarcoma viral oncogene homolog (KRAS), v-raf murine sarcoma viral oncogene homolog B1 (BRAF) and phosphatase and tensin homolog (PTEN).

In conclusion, using microarray analysis, the present study identified 11 genes that were differentially expressed between LSCC and RSCC. This therefore provided important insights into the understanding of the molecular genetic basis for the different biological behaviors that are evident between LSCC and RSCC.

## Figures and Tables

**Table I.** Baseline characteristics of patients.

Characteristic	LSCC (n=50), n (%)	RSCC (n=50), n (%)	P-value
Age (years)			
≤50	15 (30)	21 (42)	0.21
>50	35 (70)	29 (58)	
Gender			
Male	31 (62)	27 (54)	0.54
Female	19 (38)	23 (46)	
Tumor cell differentiation			
High	17 (34)	18 (36)	0.57
Intermediate	22 (44)	25 (50)	
Low	11 (22)	7 (14)	
Lymph node metastasis			
Yes	26 (52)	21(42)	0.42
No	24 (48)	29 (58)	
Liver metastasis			
Yes	11 (22)	7 (14)	0.30
No	39 (78)	43 (86)	
Tumor diameter (cm)			
≤5	22 (44)	17 (34)	0.31
>5	28 (56)	33 (66)	
Tumor invasion			
Within muscle	14 (28)	21 (42)	0.14
Serosa and further	36 (72)	29 (58)	
TNM stage			
I and II	11 (22)	16 (32)	0.26
III and IV	39 (78)	34 (68)	

LSCC, left-sided colon carcinoma; RSCC, right-sided colon carcinoma.

**Table II.** Differentially expressed genes in LSCC and RSCC identified by microarray analysis.

A, Upregulated genes in LSCC and RSCC					

GB accession	Name	Score	Fold change	Chromosome	GO biological process

U40343	*CDKN2D*	−2.456	0.347	19	Cytoplasm
BG110199	*LDHB*	4.747	3.562	12	Energy pathways
BX537504	*PI3KC2α*	2.432	3.643	6	Humoral defense mechanism
X87241	*FAT*	2.343	3.090	4	Development
L11329	*DUSP2*	2.076	2.567	2	Macromolecule metabolism

B, Downregulated genes in LSCC and RSCC					

GB accession	Name	Score	Fold change	Chromosome	GO biological process

Y12653	*UBD*	2.785	4.926	6	Organismal movement
AJ276469	*C20orf35*	−2.456	0.444	20	Cell growth and/or maintenance
AB037848	*SYT13*	−2.322	0.200	11	Coated vesicle
AK056548	*ZNF560*	−2.275	0.488	19	Transcription, DNA-dependent
AK093730	*PLEKHB2*	−2.045	0.490	2	-
D84239	*FCGBP*	−2.026	0.187	19	-

LSCC, left-sided colon carcioma; RSCC, right-sided colon carcinoma; GO, gene ontology; *CDKN2D*, cyclin-dependent kinase 4 inhibitor D; *LDHB*, L-lactate dehydrogenase B chain; *PI3KC2α*, phosphatidylinositol-4-phosphate-3-kinase C2 domain-containing subunit α; *FAT*, protocadherin fat 1; *UBD*, *DUSP2*, dual specificity protein phosphatase 2; ubiquitin D; *C20orf35*, casein kinase-1 binding protein; *SYT13*, synaptotagmin-13; *ZNF560*, zinc finger protein 560; *PLEKHB2*, pleckstrin homology domain-containing family B member 2; *FCGBP*, IgGFc-binding protein.

**Table III.** Immunohistochemical detection of UBD and CDKN2D expression in LSCC and RSCC.

	n	CDKN2D		P-value	UBD		P-value
	
		Positive, n	Positive rate (%)		Positive, n	Positive rate (%)	
RSCC	50	16	32	0.016	38	76	0.007
LSCC	50	28	56		25	50	

UBD, ubiquitin D; CDKN2D, cyclin-dependent kinase 4 inhibitor D; LSCC, left-sided colon carcioma; RSCC, right-sided colon carcinoma.
